# Growth of Semi-Polar (10$\bar{1}$3) AlN Film on M-Plane Sapphire with High-Temperature Nitridation by HVPE

**DOI:** 10.3390/ma14071722

**Published:** 2021-03-31

**Authors:** Xu Li, Jianyun Zhao, Ting Liu, Yong Lu, Jicai Zhang

**Affiliations:** 1 College of Mathematics and Physics, Beijing University of Chemical Technology, Beijing 100029, China; xuli@mail.buct.edu.cn (X.L.); jyzhao@mail.buct.edu.cn (J.Z.); 2 State Key Laboratory of Chemical Resource Engineering, Beijing University of Chemical Technology, Beijing 100029, China

**Keywords:** aluminum nitride, high-temperature nitridation, semi-polar, nano-hole

## Abstract

Aluminum nitride (AlN) films were grown on the m-plane sapphire by high-temperature hydride vapor phase epitaxy (HVPE). The effect of high-temperature nitridation on the quality of AlN film was studied. The high-temperature nitridation is favorable for the formation of semi-polar single (10$\bar{1}$3) orientation AlN film, the quality of which shows strong dependence on the nitridation temperature. The full width at half maximum of X-ray diffraction for (10$\bar{1}$3) AlN film was only 0.343° at the optimum nitridation temperature of 1300 °C. It is found that the nano-holes were formed on the surface of substrates by the decomposition of sapphire in the process of high-temperature nitridation, which is closely related to the quality improvement of AlN. At the critical nitridation temperature of 1300 °C, the average size of the nano-holes is about 70 nm, which is in favor of promoting the rapid coalescence of AlN micro-grains in the early stages. However, the size of nano-holes will be enlarged with the further increase of nitridation temperature, which begins to play a negative role in the coalescence of AlN grains. As a result, the grain size will be increased and extended to the epilayer, leading to the deterioration of the AlN film.

## 1. Introduction

Wurztie AlN is the direct band gap semiconductor material with the largest band gap of 6.2 eV among the III-nitride semiconductors [1,2]. It is an ideal substrate material for ultraviolet optoelectronic devices and has huge application potential in the fields of solid-state light sources, ultraviolet sterilization and water treatment [1,3,4,5,6]. Currently, AlN-based ultraviolet light-emitting diodes (UV-LED) are mainly grown on the polar (0001) plane, which has a strong spontaneous and piezoelectric polarization electric field, leading to a decrease in the luminous efficiency of the device [7,8,9,10,11]. In contrast, the semi-polar AlN, such as (10$\bar{1}$3) and (11$\bar{2}$2) planes, has a weak piezoelectric polarization field, which can effectively improve the performance of UV-LEDs [12,13,14].

The quality of AlN film grown at low temperature is not good due to the low migration rate of Al atoms at low temperature [15,16,17,18]. In contrast, the high temperature (>1200 °C) is beneficial to promote the coalescence of AlN grains, and thus improve the quality of AlN film [6]. Moreover, some research groups have obtained high-quality semi-polar AlN in the high-temperature region, ranging from 1200 to 1650 °C [8,15,16,17,18,19,20,21]. Jo et al. obtained smooth (11$\bar{2}$2) AlN with a thickness of 2 μm by metal organic chemical vapor deposition (MOCVD) at 1500 °C [21]. Shen et al. prepared the best quality semi-polar (10$\bar{1}$3) AlN with NH_3_-free metalorganic vapor phase epitaxy (MOVPE) at a high temperature of 1650 °C [20]. Sapphire and SiC are suitable substrates for such high growth temperature [15,22]. Compared with SiC, sapphire is transparent to ultraviolet wavelengths, and large-size sapphire are cheaper [23]. Therefore, sapphire is the first choice for preparing AlN substrates and AlN-based deep ultraviolet optoelectronic devices. However, sapphire is easy to decompose in the atmosphere of NH_3_, N_2_ and H_2_ above 1200 °C [24]. Although low-temperature nitridation [19] is effective to prevent the decomposition of sapphire, there are still some reports showing that proper holes at sapphire surface can help reduce stress and achieve smooth surface of AlN film [25,26]. Furthermore, it is well-known that nitridation has a great influence on the orientation of semi-polar AlN [27]. At present, the researches of nitridation temperature on semi-polar AlN are mainly focused on the low-temperature region [19,28,29]. Whether increasing nitridation temperature will help to reduce the stress and the surface roughness of AlN film remains to be discussed. In addition, compared to MOCVD, hydride vapor phase epitaxy (HVPE) is more suitable for AlN growth with high rate and thick film [26,30]. However, up to now, there are still few reports on the growth of semi-polar AlN film by HVPE. Therefore, it is urgent and valuable to study the effect of nitridation on semi-polar AlN grown by HVPE, especially at the high-temperature region. 

In this paper, we used the HVPE to grow semi-polar AlN on m-plane sapphire substrate. Before the growth of AlN film, the sapphire substrate was modified by high-temperature nitridation. In this process, the sapphire surface decomposes to produce nano-holes due to the high-temperature nitridation. Then, the effect of nano-holes on the growth quality of semi-polar AlN film was specifically studied. 

## 2. Experimental Details

A home-made horizontal HVPE was used to grow the semi-polar (10$\bar{1}$3) AlN. Commercial 2-inch m-plane sapphire (Helios Wafer, Jiangyin, China) was used as a substrate. HCl (Linde gas, Suzhou, China) and NH_3_ (Linggas, Beijing, China) were used as input active gases. Before AlN deposition, HCl flowed over Al source to form gaseous aluminum chlorides at 550 °C. The mixture of H_2_ and N_2_ (mixed ratio of 1:1) was used as a carrier gas and the pressure was kept at 40 Torr during the growth. At first, the m-plane sapphire substrate is heated to nitridation temperature in the carrier gas and kept for 10 min in H_2_ ambient to remove the surface oxide and achieve thermal stability. Then, sapphire nitridation is performed at different temperatures (1200, 1300, 1400 and 1500 °C, respectively) for 10 min under the ambient of NH_3_ (0.5 SLM), followed by the growth of an 80 nm buffer layer at the nitridation temperature. Then, the sapphire substrate is heated to 1500 °C and a thickness of 2.9 µm AlN film is grown with a V/III ratio of 30 and a pressure of 40 Torr. A summary of growth conditions for six samples are listed in Table 1. High-resolution X-ray diffractometer (HRXRD) (Bruker, Beijing, China) is used to characterize the orientation and quality of AlN film. Scanning electron microscope (SEM) (Bruker, Beijing, China) and transmission electron microscope (TEM) (Hitachi, Shanghai, China) are used to study the interface between AlN and sapphire. Atomic force microscopy (AFM) (Bruker, Beijing, China) is used to observe the surface morphology of the buffer layer. Finally, the stress of AlN film is evaluated by Raman spectrometer (Renishaw inVia, London, England).

## 3. Results and Discussion

**Figure 1:**
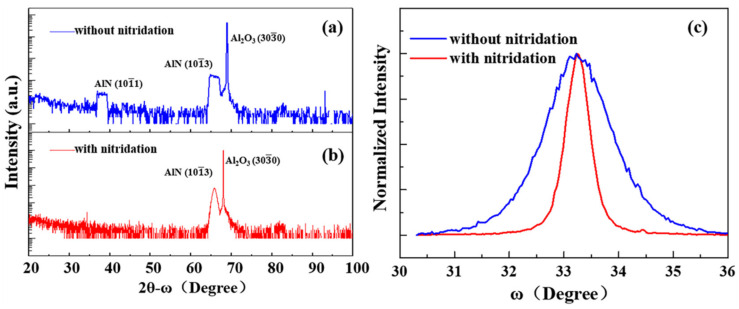
Wide 2θ-ω scans of sample (**a**) without nitridation and (**b**) with nitridation at 1400 °C. (**c**) Normalized X-ray rocking curve (XRC) of sample taken along ${\mathrm{AlN}}_{\left[1\bar{2}10\right]}$. The red and blue curves represent the sample with and without nitridation, respectively.

**Figure 2:**
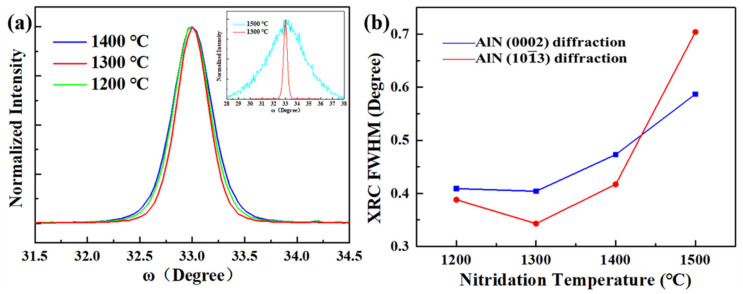
(**a**) Normalized XRC of samples with different nitridation temperatures. The green, red, blue and cyan curves are samples nitrided at 1200, 1300, 1400 and 1500 °C, respectively. (**b**) FWHM values as a function of nitridation temperature. The red and blue curves are (10$\bar{1}$3) and (0002) diffractions, respectively.

Firstly, the influence of high-temperature nitridation on the growth orientation of AlN film is studied by comparing samples S1 and S2. With the high-temperature nitridation, only the (10$\bar{1}$3) AlN diffraction peak is observed in sample S2, as shown in Figure 1b. In contrast, in sample S1 without nitridation (Figure 1a), there are both (10$\bar{1}$3) and (10$\bar{1}$1) AlN diffraction peaks. The above results indicate that high-temperature nitridation helps to achieve semi-polar single (10$\bar{1}$3) orientation AlN film. Besides, as shown in the X-ray rocking curve (XRC) (Figure 1c), the full width at half maximum (FWHM) of sample S2 is 47% smaller than S1, implying that the high-temperature nitridation can also improve the quality of the AlN film. The effect of nitridation temperature on AlN quality is then studied in detail. For samples S3, S4, S5 and S6, only the nitridation temperature is changed. From the normalized XRC taken along ${\mathrm{AlN}}_{\left[1\bar{2}10\right]}$ (Figure 2a), the sample nitrided at 1300 °C has the smallest FWHM, indicating that the quality of the AlN film is the best. However, when the nitridation temperature is higher than 1400 °C, the FWHM of the AlN film increases significantly, implying that the crystal quality is degraded. Besides, the FWHM as a function of nitridation temperature is demonstrated in Figure 2b. With the increasing nitridation temperature, on-axis (10$\bar{1}$3) FWHM and off-axis (0002) FWHM show the same trend of first decreasing and then increasing. The minimum FWHM of (10$\bar{1}$3) and (0002) both appear at 1300 °C, corresponding to 0.343° and 0.409°, respectively.

**Figure 3:**
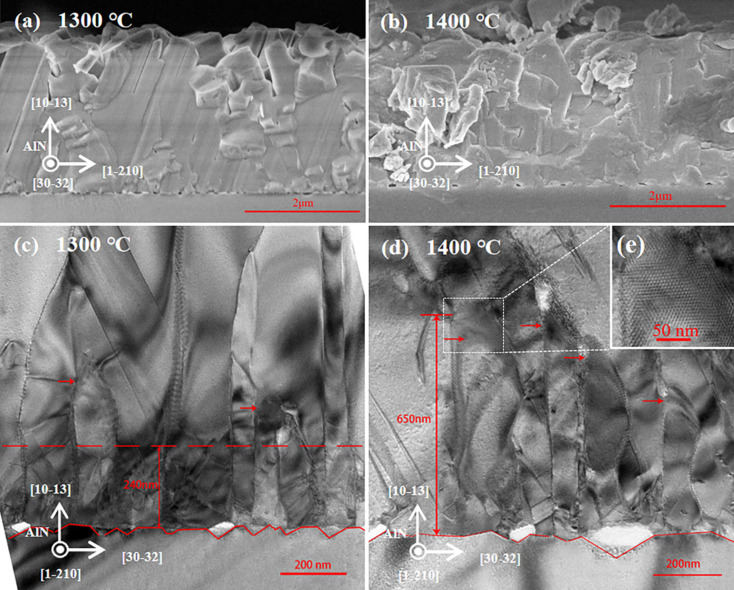
Cross-sectional scanning electron microscope (SEM) images taken along ${\mathrm{AlN}}_{\left[1\bar{2}10\right]}$ for samples (**a**) S4 and (**b**) S5. Cross-sectional transmission electron microscope (TEM) images taken along ${\mathrm{AlN}}_{\left[30\bar{3}2\right]}$ for samples (**c**) S4 and (**d**) S5. (**e**) Enlarged view of the distorted area marked by the dashed rectangle in (**d**).

In order to clarify the influence mechanism of nitridation on AlN film, the interface between sapphire and AlN is deeply investigated by SEM and TEM. From the SEM images (Figure 3a,b) taken along ${\mathrm{AlN}}_{\left[1\bar{2}10\right]}$, it is clear to see that there are many nano-holes at the interface between sapphire and AlN film. These nano-holes are formed by the decomposition of sapphire during high-temperature nitridation [25]. More specifically, the size and density of the nano-holes in samples S4 and S5 are listed in Table 2. In sample S4, the average length, height and line density of the nano-holes are 69.0, 33.3 nm and 3.8 μm^−1^, respectively. While in sample S5, the average length, height and line density of the nano-holes correspond to 92.8, 39.6 nm and 1.3 μm^−1^, respectively. Comparing with sample S5, the size of the nano-hole in sample S4 is smaller but the density is larger. In fact, higher nitridation temperature will aggravate the decomposition of sapphire, leading to the expansion of small nano-holes. As a result, those adjacent small holes may be connected to form larger ones. Therefore, the nano-holes in sample S5 become larger but the density decreases.

The morphology of nano-holes at the interface can be observed more clearly through TEM taken along ${\mathrm{AlN}}_{\left[30\bar{3}2\right]}$. In sample S4 (Figure 3c), the nano-holes on the surface of sapphire are triangular and the AlN above the large holes is also decomposed. More specifically, the average length, height and line density of the triangular nano-holes in sample S4 are 62.2, 27.4 nm and 9.0 μm^−1^, respectively. From the line density of ${\mathrm{AlN}}_{\left[30\bar{3}2\right]}$ and ${\mathrm{AlN}}_{\left[1\bar{2}10\right]}$, the surface density of holes on (10$\bar{1}$3) is calculated to be 34.2 μm^−2^. We think the nano-holes at the interface help to reduce the size of AlN grains during initial growth and promote small AlN grains’ merging within a thickness of 240 nm. Therefore, in the subsequent growth process, the length and density of the AlN grains are both reduced. At the top of the long AlN grain, there are some distortions marked by the red arrows, which are caused by the coalescence of the adjacent grains. These distorted regions will cause the deterioration of AlN film, thereby widening XRC. Similarly, the average base length, height, line density and the surface density of nano-holes on (10$\bar{1}$3) in sample S5 (Figure 3d) are determined as 74.4, 42.2 nm, 6.2 μm^−1^ and 8.1 μm^−2^, respectively. Although the density of the nano-holes is decreased with the higher nitridation temperature, the enlarged nano-holes result in the rugged surface of sapphire. As the density of nano-holes decreases, the number and height of AlN grains increase and they become difficult to merge. Besides, the distorted zone (marked by the red arrow) is significantly expanded, leading to worse AlN film. Figure 3e shows the enlarged view of the highly distorted area. In addition, the largest nano-hole can be seen in Figure 3d, the basic length of which reaches 211.5 nm. Compared with other small holes, AlN grains are more difficult to merge above this large hole, together with more chaotic orientation of AlN. As a result, we believe that large-size nano-holes are favorable for promoting coalescence of AlN grains, so they cannot improve the quality of AlN film.

**Figure 4:**
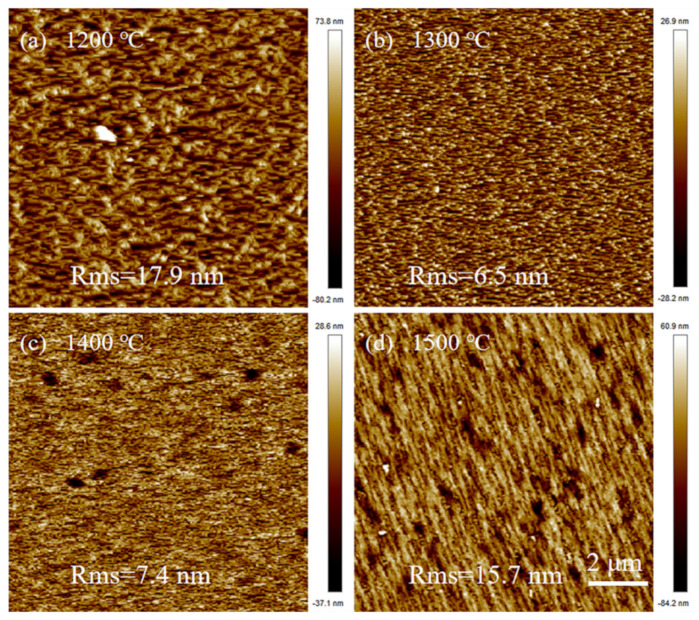
10 × 10 μm AFM image of buffer layers grown at different temperatures of (**a**) 1200 °C, (**b**) 1300 °C, (**c**) 1400 °C and (**d**) 1500 °C, respectively.

Furthermore, the coalescence of AlN grains will influence the surface morphology of the AlN buffer layer. Figure 4 shows the AFM images of buffer layers for four samples with different nitridation temperatures. When the nitridation temperature is 1200 °C (Figure 4a), the surface of the buffer layer is the roughest. This indicates that the lateral migration of Al atoms at 1200 °C is insufficient, so the growth of the buffer layer is not fully converted from three-dimensional (3D) mode to two-dimensional (2D) mode. When nitriding at 1300 °C, the surface of the buffer layer is the smoothest (Figure 4b), with a roughness of only 6.5 nm. As the nitridation temperature increases, some pits are observed in the buffer layer, leading to the increase in roughness. Actually, the pits in the buffer layer are related with the large-sized nano-holes at the sapphire surface. For the AlN above the large nano-holes, the high density of defects causes AlN to be easily decomposed, thereby generating pits in the buffer layer. Moreover, the higher the nitridation temperature (Figure 4d), the more pits, so the roughness of the buffer layer becomes larger.

**Figure 5:**
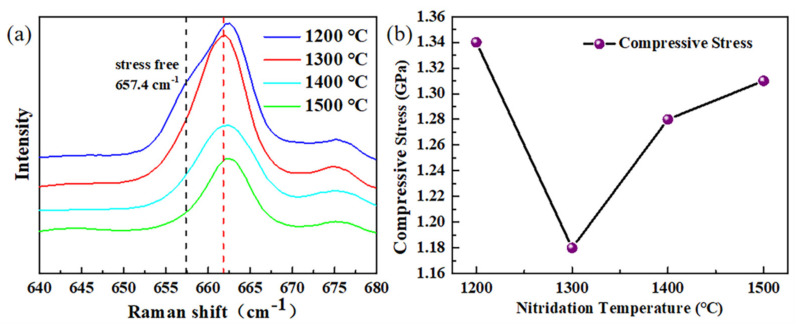
(**a**) Raman spectra for AlN samples grown at different nitridation temperatures. The blue, red, cyan and green curves correspond to 1200, 1300, 1400 and 1500 °C, respectively. (**b**) The compressive stress of AlN film as a function of the nitridation temperature.

We also evaluated the residual stress of the semi-polar (10$\bar{1}$3) AlN film with different nitridation temperatures. In Raman measurement, the $E_{2}^{\mathrm{high}}$ peak of AlN can reflect the residual stress of AlN film, the value of which under stress-free condition is 657.4 cm^−1^ [31]. As shown in Figure 5a, the $E_{2}^{\mathrm{high}}$ peak of all samples is larger than 661 cm^−1^, indicating that the AlN film is in compressed state. More importantly, the residual stress of AlN films can be obtained through the following equation:

(1) $$\sigma =\frac{\Delta \omega }{2\tilde{a}}$$

where $\tilde{a}$ is constant Phonon Deformation Potential (PDP) parameters, Δω is the frequency shift of $E_{2}^{high}$ and σ denotes stress [32,33]. Taking $\tilde{a}$ as 3.74 cm^−1^GPa^−1^ [32], the residual stress of samples with different nitridation temperatures are calculated. Figure 5b shows that all samples are under compressive stress. Moreover, the samples nitrided at 1300 °C have the least stress, of 1.18 GPa, and the highest density of nano-holes at the surface of sapphire. Therefore, it is reasonable to consider that the high density of nano-holes at the surface of substrate is favorable to the stress release of AlN film. In the future, further research will be carried out to reduce the residual stress of the semi-polar AlN film.

## 4. Conclusions

In conclusion, high-temperature nitridation of sapphire helps to obtain a single (10$\bar{1}$3) orientation AlN film. The optimal nitridation temperature is 1300 °C, at which AlN film has the minimum FWHM of 0.343°. The improvement of AlN quality is attributed to the nano-holes produced by the decomposition of the sapphire substrate under high-temperature nitridation. The high-density nano-holes of less than 100 nm promote AlN grains’ merging at the initial growth, thus improving the quality of AlN film. However, the AlN grains are difficult to merge when the nano-holes become larger under higher nitridation temperature, leading to the deterioration of AlN film.

## Figures and Tables

**Table 1:** Summary of nitridation temperature, V/III ratio of buffer layers and X-ray diffraction peaks for AlN film grown on m-plane sapphire substrate.

Samples	S1	S2	S3	S4	S5	S6
Nitridation (°C)	No	1400	1200	1300	1400	1500
V/III ratio of buffer layers	90	90	150	150	150	150
X-ray diffraction Peaks	$\left(10\bar{1}1\right)$ $\left(10\bar{1}3\right)$	$\left(10\bar{1}3\right)$	$\left(10\bar{1}3\right)$	$\left(10\bar{1}3\right)$	$\left(10\bar{1}3\right)$	$\left(10\bar{1}3\right)$

**Table 2:** Summary on the size and density of nano-holes at the interface between sapphire and AlN film.

Samples	Direction	Average Length (nm)	Average Height (nm)	Line Density (μm^−1^)	Surface Density (μm^−2^)
**S4** (Nitridation at 1300 °C)	${\mathrm{AlN}}_{\left[1\bar{2}10\right]}$	69.0	33.3	3.8	34.2
	${\mathrm{AlN}}_{\left[30\bar{3}2\right]}$	69.2	27.4	9.0	
**S5** (Nitridation at 1400 °C)	${\mathrm{AlN}}_{\left[1\bar{2}10\right]}$	92.8	39.6	1.3	8.1
	${\mathrm{AlN}}_{\left[30\bar{3}2\right]}$	74.4	42.2	6.2	

## Data Availability

Data sharing is not applicable to this article.
